# Dynamic connectedness between stock markets in the presence of the COVID-19 pandemic: does economic policy uncertainty matter?

**DOI:** 10.1186/s40854-021-00227-3

**Published:** 2021-03-01

**Authors:** Manel Youssef, Khaled Mokni, Ahdi Noomen Ajmi

**Affiliations:** 1 College of Business Administration, Northern Border University, Arar, 91431 Saudi Arabia; 2 Institut Supérieur de Gestion de Gabès, Gabès University, 6002 Gabès, Tunisia; 3 Department of Business Administration, College of Science and Humanities in Slayel, Prince Sattam Bin Abdulaziz University, Al-Kharj, Saudi Arabia; 4 ESC de Tunis, Manouba University, Manouba, Tunisia

**Keywords:** Stock markets, Dynamic connectedness, COVID-19 pandemic, Economic policy uncertainty, TVP-VAR model, C22, I15, I18, D53

## Abstract

This study investigates the dynamic connectedness between stock indices and the effect of economic policy uncertainty (EPU) in eight countries where COVID-19 was most widespread (China, Italy, France, Germany, Spain, Russia, the US, and the UK) by implementing the time-varying VAR (TVP-VAR) model for daily data over the period spanning from 01/01/2015 to 05/18/2020. Results showed that stock markets were highly connected during the entire period, but the dynamic spillovers reached unprecedented heights during the COVID-19 pandemic in the first quarter of 2020. Moreover, we found that the European stock markets (except Italy) transmitted more spillovers to all other stock markets than they received, primarily during the COVID-19 outbreak. Further analysis using a nonlinear framework showed that the dynamic connectedness was more pronounced for negative than for positive returns. Also, findings showed that the direction of the EPU effect on net connectedness changed during the pandemic onset, indicating that information spillovers from a given market may signal either good or bad news for other markets, depending on the prevailing economic situation. These results have important implications for individual investors, portfolio managers, policymakers, investment banks, and central banks.

## Introduction

Academics, policymakers, and investors have heated discussions over analyzing the connectedness between financial markets, but this analysis was recently reinforced by mathematical and econometric tool development. These tools increased its importance by providing a comprehensive picture of market risk, credit risk, and macroeconomic and system risk evaluation (Gong et al. 2019) to support better decision-making (Kou et al. 2014). Furthermore, analyzing connectedness between financial assets, especially stocks, is also essential for investors because it helps them to assess and make suitable decisions about international portfolio diversification opportunities.

Accordingly, several studies investigating relationships between financial markets reported that the degree of connectedness between these markets is subject to financial stress and crises, such as the global financial crisis (GFC), the European debt crisis (Jondeau and Rockinger 2006; Mokni and Mansouri 2017) and the health crisis driven by the COVID-19 outbreak in 2020 (Azimli 2020; Cepoi 2020; Topcu 2020; Sharif et al. 2020). The rapidly expanding pandemic has had enormous negative effects on financial markets worldwide, creating an unprecedented level of risk that caused investors to suffer significant losses over a very short period (Zhang et al. 2020a, b, c). In fact, this crisis resulted in depreciated stock indices, especially in countries with high infection rates. Moreover, the degree of uncertainty increased following the news of China’s first case at the end of 2019, especially after the World Health Organization declared the coronavirus outbreak a global pandemic on 11 March 2020.

Given that stock markets worldwide are directly or indirectly linked to economic systems and strong financial integration, they suffered enormous losses after precautionary measures were implemented by most countries. Therefore, the implications regarding the pandemic’s effect on stock markets exposed serious questions about the market’s dynamics and connectedness.

Azimli (2020) reported that the COVID-19 pandemic impacts the stock market through two channels. First, the high level of economic policy uncertainty stemming from the pandemic’s spreading patterns and the unknown future situation regarding COVID-19 lead to low cash flow expectations, resulting in stock market depreciation. Second, halting industrial, tourism, aviation, and other sectors directly affects the stock index by depreciating the related stocks. This degradation negatively impacts macroeconomics, such as investment and consumption patterns (Azimli 2020).

In addition to crisis and stress conditions, the international stock markets’ degree of spillover coupled with economic policy uncertainty (EPU) influence the dynamics and even change the market’s direction. In this context, the EPU index, developed by Baker et al. (2016), influences stock market returns (Antonakakis et al. 2013; Arouri et al. 2016; Christou et al. 2017; Guo et al. 2018; Hu et al. 2018; Phan et al. 2018; Xiong et al. 2018; He et al. 2020) and volatility (Yu et al. 2018; Yu and Song 2018; Mei et al. 2018; Balcilar et al. 2019; Wang et al. 2020). Therefore, a recent strand of literature focused on EPU’s effect on relationships between financial assets, including relationships between stock markets (Li and Peng 2017), bonds and stocks (Fang et al. 2017; Li et al. 2015), commodity and stock markets (Fang et al. 2018; Badshah et al. 2019), and Bitcoin and conventional assets (Matkovskyy et al. 2020). Nearly all these studies reported evidence of a negative impact of EPU on the co-movement between these variables, and, in some cases, highlighted a significant portfolio implication related to EPU (Badshah et al. 2019).

This study investigates the dynamic connectedness between stock indices in countries with widespread coronavirus infections. We also examined the effect of EPU on this connectedness before and during the COVID-19 pandemic crisis. Dynamic connectedness was measured using Antonakakis and Gabauer’s (2017) method, which combines the time-varying VAR (TVP-VAR) model with Diebold and Yilmaz’s (2014) popular model. We chose the TVP-VAR model to extract the connectedness indices because it has some advantages over the rolling-window-based VAR. First, it overcomes the arbitrary set of window size, which leads to very erratic or flattened parameters. Second, unlike the rolling-window-based VAR, the TVP-VAR model is estimated based on the Kalman filter procedure; therefore, valuable observations are not lost when estimating the model’s time-varying parameters. Third, this model is not sensitive to outliers (Antonakakis and Gabauer 2017; Antonakakis et al. 2018; Gabauer and Gupta 2018; Korobilis and Yilmaz 2018). Our use of the TVP-VAR model to extract the connectedness indices is consistent with Korobilis and Yilmaz’s (2018) similar approach, which used Bayesian VAR approaches to overcome the rolling-window-based VAR limitations.

This study contributes to the existing literature by investigating the dynamic connectedness between several international stock markets in countries most affected by COVID-19 during a sample period that incorporated the health crisis in symmetric and asymmetric frameworks. Moreover, we investigated economic policy uncertainty’s role in driving the dynamic connectedness between these markets before and during the COVID-19 pandemic crisis. This analysis controlled for potential changes in the effect during the COVID-19 period compared to the pre-outbreak period.

The remainder of this paper is organized as follows. The second section presents the literature review. The third section presents the data and the methodology employed. Section "Results and discussions" presents the results and discussion. Finally, section "Conclusion and policy implications" concludes the paper.

## Literature review

During the last two decades, financial crises led to renewed interest in examining connectedness, contagion, and correlations between stock markets using different econometric techniques. Overall, financial crises have increased market connectedness across stock markets. For example, Madaleno and Pinho (2012) explored stock market contagion using a continuous-time wavelet method (Coherence Morlet Wavelet) that considered financial crisis episodes and found a significant time evolution, especially related to financial crises that occurred at different periods. Kim et al. (2015) used multivariate GARCH models to investigate the impact of the US financial crisis on spillover effects between five emerging Asian countries and the US stock markets. They found clear evidence of financial contagion around the collapse of Lehman Brothers in September 2008. Li and Giles (2015) investigated both shock and volatility spillovers in long‐run and short‐run periods in the US, Japan, and six emerging stock markets (China, India, Indonesia, Malaysia, the Philippines, and Thailand) using the asymmetric BEEK-MGARCH model. They reported intensified integration between developed and emerging stock markets during financial crises.

Also, Morana and Beltratti (2008) investigated co-movements in four international stock markets (US, UK, Germany, and Japan) over the period between 1973 and 2004. They showed that integrating these different stock markets induced rising co-movements in prices, returns, correlation, and volatility. Menezes and Dionísio (2011) used a VECM model under structural breaks to investigate the long-run co-movements and globalization in G7 stock markets and found a significant long-run causal relationship across the process of G7 market integration, driven in general by the US stock market. Tsai (2014) investigated the spillover effect in the main five stock markets (US, UK, Germany, Japan, and France) and found a net spillover effect in the US stock market during the subprime mortgage crisis and Lehman Brothers bankruptcy from 2007 to 2008. He also showed that the fear index was a driving force behind the increased correlation between markets. Using the DCC-GARCH methodology, Ahmad et al. (2014) investigated the contagion effects of US and GIPSI stock markets on seven Eurozone and six non-Eurozone stock markets. They reported that among the GIPSI markets, Italy, Ireland, Portugal, and Spain were the most contagious for Eurozone and non-Eurozone markets during the Eurozone crisis period. However, France, Belgium, Austria, and Germany were strongly affected by the financial contagion shocks in the Eurozone. Výrost et al. (2015) used Granger causality networks to investigate the network between 20 developed stock markets. They found that preferential attachment between stock markets and the degree of connectedness positively affected spillover effects. Maghyereh et al. (2015) applied Diebold and Yilmaz’s (2012) methodology to investigate equity returns and volatility co-movement between the MENA group and US stock markets before and after the global financial crisis. They reported that the relationship with the US stock market was very weak in the pre-crisis period but bounded to a high level after the global financial crisis. Baruník et al. (2016) extended Diebold and Yilmaz’s (2012) methodology by allowing for negative and positive changes to quantify asymmetries in volatility spillover and found that connectedness across the US intra–market rose steeply during the financial crisis.

More recently, Wang et al. (2017) used multiscale correlation to examine contagion from the US to the BRIC and the G7 (except for Japan) during the global financial crisis. They reported that the stock markets' contagion during the global financial crisis was dependent on both the recipient country and the time scale. Jiang et al. (2017) applied the VAR model and Granger causality tests to explore the recent financial crisis' impact on six major stock markets (US, Britain, Germany, Japan, China, and Hong Kong). Results showed that the financial crisis boosted the interdependence correlation of global stock markets. Mokni and Mansouri (2017) examined relationships between major international stock markets using a long memory GARCH-copula model and reported that the dependence structure rose during the European debt and global financial crises. Manopimoke et al. (2018) investigated the dynamic connectedness between Asian emerging markets and other international markets using a generalized vector autoregressive (VAR) model. They concluded that international stock markets were integrated, with a rising trend following the Asian financial crisis and a more important trend of increasing during the global financial crisis. Mensi et al. (2018) applied Diebold and Yilmaz’s (2012, 2014) methodology by implementing static and rolling-window methods to examine the connectedness between regional, global, and GIPSI stock markets and reported that the financial contagion effect increased during the crisis. Zhou et al. (2018) applied a CEEMDAN wavelet model to examine the contagion effect among stock markets (Asia, Europe, and America) and reported that shocks caused by irregular events and extreme events could be transmitted between different stock markets. Gong et al. (2019) applied the transfer entropy method to investigate the network connectedness of global stock markets, and suggested that total network connectedness rises during the crisis. Kang et al. (2019) used a dynamic equi-correlation (DECO) model, and Diebold and Yilmaz’s (2012) spillover index to investigate the dynamic spillover effects between ASEAN and world stock markets. They reported directional spillovers for each of the markets and increased return and volatility spillovers during financial crises. Su (2020) examined the volatility spillover behaviors in G7 stock markets using a spectral representation of variance decomposition to distinguish between short, medium, and long-term volatility spillover components. He reported crisis sensitivity of the volatility spillovers across G7 stock markets.

Many studies have investigated the impacts of the virus outbreak on stock market performance. Delisle (2003) proposed that the cost of the 2003 SARS outbreak resulted in losses as high as those resulting from Asia’s financial crisis, estimated as $3 trillion in GDP and $2 trillion in financial market equity. Nippani and Washer (2004) examined the effect of SARS on Canada, China, and the specific administrative regions of Hong Kong, Indonesia, Singapore, the Philippines, Vietnam, and Thailand. They concluded that SARS only affected China’s and Vietnam’s stock markets. In the same context, Lee and McKibbin (2004) evaluated the global economic impacts of severe acute respiratory syndrome (SARS). They state the effect of the SARS epidemic on global human society was severe, not only because the disease spread through countries rapidly from global travel, but also, with financial integration and globalization, any economic shock to one country spreads rapidly to others. Macciocchi et al. (2016) studied the short-term economic impact of the Zika virus outbreak on Brazil, Argentina, and Mexico, and showed that, except for Brazil, the market indices of these three Latin American and Caribbean Countries (LCR) did not show large negative returns the day after each shock. Marinc (2016) investigated whether the geographical proximity of information disseminated by the 2014 Ebola outbreak, coupled with widespread media coverage, affected US asset prices. Chen et al. (2018) analyzed the impact of the SARS epidemic on China’s long-term relationship with four Asian stock markets. Their findings supported a time-varying co-integration relationship in aggregate stock price indices. They also found that the SARS epidemic weakened China’s long-term relationship with the four markets.

Since its appearance, COVID-19′s effects are frequently compared with the 2008 global financial crisis. In recent studies, the effects of the COVID-19 pandemic on stock markets were exanimated using different approaches. For example, Salisu and Vo (2020) investigated the relevance of health news obtained through Google searches in predicting stock returns using data from the top-20 affected countries and the countries reporting the most deaths. They found that including health-related information in stock valuation improved forecast accuracy. Forecast performance was also improved by adjusting for macroeconomic factors and accounting for the “asymmetry” effect of good and bad health news. Al-Awadhi et al. (2020) examined COVID-19′s effect on the Chinese stock market by applying panel testing while controlling for firm-specific characteristics. They found significant negative effects on stock returns caused by COVID-19 across all companies.

Sharif et al. (2020) used wavelet-based Granger causality and coherence wavelet tests to investigate the time–frequency relationship between oil price, COVID-19 outbreak, economic uncertainty, geopolitical risk, and the US stock market. They showed that the effect of COVID-19 on geopolitical risk was substantially higher than the effect on US economic uncertainty. Using the DCC-GARCH methodology, Corbet et al. (2020a) examined the effects of the term “corona” on stocks' behavior during the COVID-19 pandemic. They showed a negative effect for companies with names related to the coronavirus pandemic.

Azimli (2020) applied quantile regression to investigate the COVID-19 pandemic’s effect on the degree and structure of risk-return dependence in the US and found an increase in dependence among returns and market portfolios in the higher quantiles. Corbet et al. (2020b) investigated contagion between Chinese stock markets during the COVID-19 pandemic and found that COVID-19 had a significant strong positive impact on the volatility of the Shanghai and Shenzhen Stock Exchanges. They also reported a strong positive correlation between WTI and Chinese stock markets. In the same context, Ashraf (2020) investigated stock markets’ reaction to COVID-19. Using daily COVID-19 confirmed cases and deaths, he found that stock markets responded negatively to increases in COVID-19 confirmed cases. Liu et al. (2020) and Khan et al. (2020) used econometric models to evaluate the short-term impact of the coronavirus outbreak on major affected countries’ stock market indices. Results showed that stock markets fell quickly after the virus outbreak. Moreover, their findings supported the adverse effects of confirmed COVID-19 cases on abnormal returns through an effective channel by identifying investors’ pessimistic sentiment regarding future returns and fear of uncertainties. Similarly, Zhang et al. (2020a, b, c) investigated the general pattern of country-specific risks and system risks in the global financial markets under the COVID-19 pandemic. They also analyzed the potential consequences of policy interventions, such us the US decision to implement a zero-percent interest rate and unlimited quantitative easing. They found evidence of increasing global market volatility because of the outbreak, and that global stock market linkage displayed different patterns before and after the pandemic announcement. Moreover, they found that policy responses may have generated further uncertainties in the global financial markets.

So et al. (2020) studied the impact of the COVID-19 pandemic on the Hong Kong stock market connectedness. Using dynamic financial networks based on stock return correlations, they found that both network density and clustering were higher in the partial correlation networks during the COVID-19 pandemic, implying increased network connectedness in the financial networks and equally substantial increases in systemic risk during the outbreak.

In addition to health disasters and financial crises, stock markets are also affected by economic policy uncertainty (EPU). Several studies reported that EPU had a large impact on stock market dynamics relationships. For instance, Antonakakis et al. (2013) examined correlations between stock market returns, implied volatility, and policy uncertainty in a time-varying framework and found that an increase in the volatility of policy uncertainty dampened stock market returns. Arouri et al. (2016) studied the impact of EPU on the US stock market between 1900 and 2014 and reported a significant negative relationship between policy uncertainty and stock returns, where EPU’s effect on stock returns was stronger and more persistent during extreme volatility periods. Using a VAR panel model, Christou et al. (2017) investigated the role of economic policy uncertainty (EPU) on stock market returns for six countries (Australia, Canada, China, Japan, Korea, and the US). The results suggested that increasing EPU levels affected stock market returns. More recently, Wang et al. (2020) analyzed spillover effects between EPU and stock market realized volatility (RV). Results showed that RV was a net receiver of uncertainty shocks, with higher effects from US EPU than from Chinese EPU. Balcilar et al. (2019) investigated EPU’s role in predicting stock returns’ volatility in Hong Kong, Malaysia, and South Korea. Based on the nonparametric Granger causality in quantiles model, results showed strong evidence of EPU causing stock return volatility in Malaysia and both returns and volatility in certain parts of South Korea’s conditional distributions.

This large volume of literature supports the conclusion that stock market dynamics and relationships depend strongly on stress conditions, public health crises, and increased uncertainty. The current study examined stock market relationships amid the COVID-19 pandemic. We expected that relationships between markets changed during the COVID-19 period compared to the prior normal period. In addition, we anticipated that EPU effects on stock market connectedness were substantially changed during this health crisis period.

## Data and econometric framework

### Data

The abnormal situation resulting from COVID-19 offered an opportunity to assess the pandemic's impact on the most affected nations' stock markets owing to this unforeseen and feared disease. This study investigated the impact of the COVID-19 pandemic on the connectedness between the major affected nations’ stock markets as measured by their leading stock indices. We used data from daily stock indices in China (SSE), France (CAC40), Germany (DAX30), Italy (FTSE MIB), Russia (RTSI), Spain (IBEX), the UK (FTSE100), and the US (S&P500). The data span from 01/01/2015 to 05/18/2020, including the beginning of the COVID-19 pandemic in 2020. To investigate EPU’s role in driving the connectedness between the examined stock markets, we also considered the EPU news-based index developed by Baker et al. (2016).^1^ The stock market data were sourced from Datastream, and the EPU index was obtained from the website https://www.policyuncertainty.com. We calculated the return series as the log-difference of the indices and the EPU index.

**Fig. 1:**
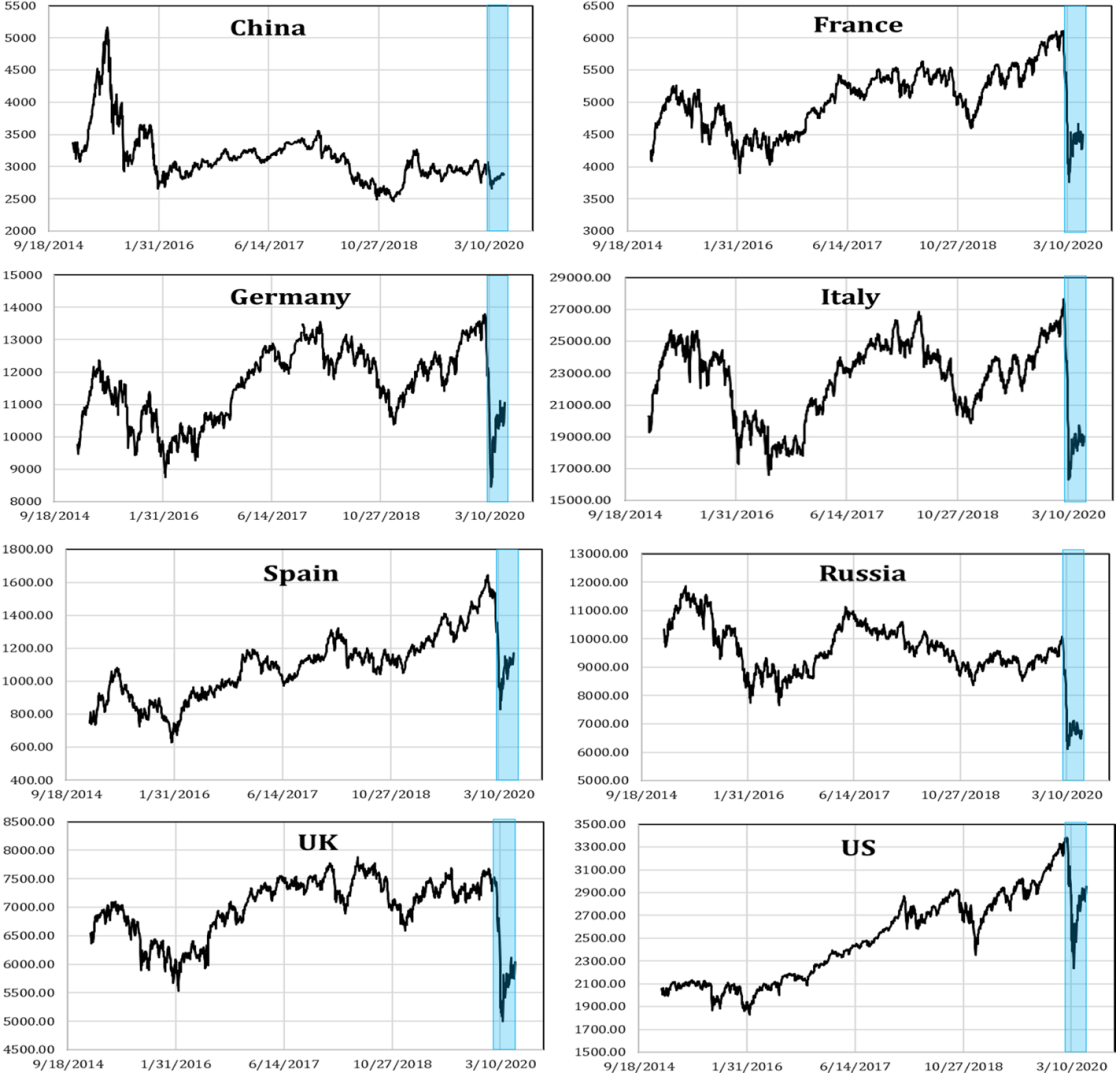
Daily spot prices of stock markets considered for the period running from 01/01/2015 to 05/18/2020. The shaded area indicates the period of the COVID-19 pandemic

Figure 1 depicts the daily spot prices of the examined countries’ stock market indices. As shown, all the examined stock market indices exhibited a sudden and abnormal decline near the onset of the COVID-19 pandemic. The patterns show that the SSE index registered a weak decline from 3000 to around 2500 points. However, the European stock markets and the American and Russian stock markets were the most affected by the spread of the new infectious shock and registered an unprecedented shutdown of their indices’ spot prices. In Italy, just before the pandemic outbreak, the index was over 27,000 points but fell to under 17,000 points with the pandemic onset. For the US, the index daily spot prices reached a peak above 3300 points before the COVID-19 pandemic outbreak and fell to under 2300 points when the new pandemic was announced. These results highlight the rapid responses of worldwide stock markets to bad news related to the COVID-19 pandemic outbreak, which increased risk and uncertainty globally and affected investors’ sentiments and decisions, which in turn affected stock market prices.

Table 1 reports descriptive statistics on the log-returns of the examined markets’ stock indices. China, Spain, and the UK presented the lowest return averages, while the US, France, Russia, and Italy exhibited the highest average returns; these countries exhibited close mean returns at around 0.03. Also, Russia had the riskiest stock market, as measured by the variance, followed by China and Italy. Whereas the British stock market presented the lowest risk average, followed by the US and France stock markets. All countries were skewed to the left, as indicated by the significant negative values of the skewness. Also, we observed that all returns series were characterized by excess kurtosis, suggesting a leptokurtic distribution with fat tails. The null hypothesis of normality was rejected at the 1% level for all series, as indicated by the Jarque–Bera test.

**Table 1:** Descriptive statistics and preliminary tests on the data

	China	France	Germany	Italy	Russia	Spain	UK	U.S
Mean	0.002	0.034	0.029	0.030	0.032	0.000	0.010	0.035
Variance	2.193	1.546	1.649	2.082	3.398	1.739	1.129	1.375
Skewness	−1.139***	−1.344***	−0.871***	−2.126***	−0.849***	−2.202***	−0.988***	−1.087***
Kurtosis	7.036***	15.402***	14.281***	26.074***	8.802***	25.231***	17.225***	24.133***
JB *p* value	0.000	0.000	0.000	0.000	0.000	0.000	0.000	0.000
ERS	−11.138***	−5.272***	−10.716***	−8.481***	−2.908***	−3.252***	−3.708***	−3.031***
ADF	−34.934***	−24.170***	−23.615***	−24.716***	−37.991***	−24.211***	−36.264***	−10.991***
PP	−34.937***	−36.582***	−36.375***	−39.106***	−38.052***	−37.805***	−36.263***	−43.869***
Q(20)	22.303***	16.165*	14.064	27.328***	13.041	21.215***	26.420***	116.391***
Q2(20)	101.408***	20.766**	98.182***	1.716	188.782***	6.79	70.659***	301.223***
LM(20)	119.824***	193.533***	236.167***	39.210***	208.550***	89.173***	237.360***	480.568***
*Correlation matrix*								
China	1							
France	0.219	1						
Germany	0.207	0.942	1					
Italy	0.15	0.866	0.845	1				
Russia	0.183	0.552	0.515	0.493	1			
Spain	0.179	0.889	0.851	0.888	0.521	1		
UK	0.223	0.866	0.829	0.753	0.583	0.784	1	
US	0.179	0.605	0.583	0.551	0.384	0.581	0.601	1

This table reports the descriptive statistics of the considered stock market returns. J.B. is the Jarque–Bera normality test statistics. ERP, ADF, and PP denote the Elliot-Rothenberg-Stock, Augmented Dicky-Fuller, and Phillip-Perron unit root test, respectively. Q(10) and Q2(10) are the Ljung-Box tests for 20th order serial correlations for returns and squared returns, respectively. L.M. (20) is the L.M. heteroscedasticity test at order 20. (***), (**), and (*) indicate the statistical significance, respectively, at 1%, 5%, and 10% levels

To test for the existence of unit root in the return series, we conducted the Elliot- Rothenberg-Stock (ERS), Augmented Dicky-Fuller (ADF), and Phillips-Perron (PP) tests, which allow testing the null hypothesis of a unit root versus the alternative of no unit root in the stock returns series. Results presented in Table 1 show that the null hypothesis was rejected at the 1% significance level for all series and, therefore, integrated of order zero I(0). Moreover, the results suggested that all series (except Spain and Italy) were autocorrelated and exhibited ARCH errors, supporting TVP-VAR model use with time-varying covariances. All examined stock markets were correlated during the sample period. The Chinese stock market exhibited the lowest correlation with all other stock markets, while all other stock markets were highly correlated. The strongest correlation was observed between the French and German stock markets by a value of 0.942, because of the strong integration of European markets (Jondeau and Rockinger 2006; Mokni and Mansouri 2017).

### Methodology

#### TVP-VAR-based dynamic connectedness approach

To explore the time-varying connectedness between international stock markets during a specified period, including the COVID-19 pandemic, we followed Antonakakis and Gabauer’s (2017) and Antonakakis et al.’s (2019) methodology, which employs Koop and Korobilis’s (2014) TVP-VAR methodology, combined with Diebold and Yılmaz’s (2014) connectedness approach.

Let $Y_{t}$ be a $\left( {N \times 1} \right)$ vector of N stock market returns. The TVP-VAR model can be represented by the following set of equations:

1 $$Y_{t} = \Phi_{t} Y_{t - 1} + u_{t} ; u_{t} \backslash \Omega_{t - 1} \sim N\left( {0,S_{t} } \right)$$

2 $$\Phi_{t} = \Phi_{t - 1} + v_{t} ; v_{t} \backslash \Omega_{t - 1} \sim N\left( {0,R_{t} } \right)$$

where ${\Omega }_{t - 1}$ denotes the set of information available at $t - 1$. $Y_{t - 1}$ is a ($Np \times 1)$ lagged vector of the dependent variables. ${\Phi }_{t}$ is an ($N \times Np)$ matrix of coefficients, which is supposed to be time-varying. $u_{t}$ and $v_{t}$ are two ($N \times 1)$ vectors of the error terms. $S_{t}$ and $R_{t}$ are ($N \times N)$ and ($Np \times Np)$ time-varying variance–covariance matrices of the error terms $u_{t}$ and $v_{t}$, respectively.

After estimating the time-varying parameters and variances using the TVP-VAR, this model estimates Diebold and Yılmaz’s (2014) generalized connectedness procedure based on the generalized impulse response functions (GIRF) and the generalized forecast error variance decompositions (GFEVD) after transforming the VAR to its vector moving average (Koop et al. 1996; Pesaran and Shin 1998). Therefore Eq. (1) can be transformed as follows:

3 $$Y_{t} = \Phi_{t} Y_{t - 1} + u_{t} = A_{t} u_{t}$$

where $$A_{t} = \left( {\begin{array}{*{20}c} {\begin{array}{*{20}c} {A_{1,t} } & {A_{2,t} ,} \\ \end{array} } & {\begin{array}{*{20}c} \ldots & {A_{p,t} } \\ \end{array} } \\ \end{array} } \right)^{\prime}$$ is an $\left( {N \times N} \right)$ matrix of parameters verifying $A_{i,t} = \mathop \sum \limits_{k = 1}^{p} {\Phi }_{1,t} A_{i - k,t} if i \ne 0$, and $I_{N}$ otherwise. Therefore, the generalized impulse response functions (GIRF) define the responses of all variables following a shock in variable *i*.

Antonakakis and Gabauer (2017) computed the differences between a J-step-ahead forecast once where variable $i$ is shocked and once where variable $i$ is not shocked. Formally, let J be the forecast horizon and $\delta_{j,t}$ be the selection vector equal to 1 on the $j^{th}$ position, and 0 otherwise, the GIRF, denoted by ${\Psi }_{j,t}^{g} \left( J \right),$ can be calculated by:

4 $$GIRF\left( {J,\delta_{j,t} , {\Omega }_{t - 1} } \right) = E\left( {Y_{t + J} \backslash u_{j,t} = \delta_{j,t} , {\Omega }_{t - 1} } \right) - E\left( {Y_{t + J} \backslash {\Omega }_{t - 1} } \right)$$

5 $${\Psi }_{j,t}^{g} \left( J \right) = S_{jj,t}^{{ - \frac{1}{2}}} A_{J,t} S_{t} u_{j,t}$$

Also, the GFEVD for the horizon J, denoted by ${\Pi }_{j,t}^{g} \left( J \right)$, can be calculated by:

6 $$\Pi_{j,t}^{g} \left( J \right) = \frac{{\mathop \sum \nolimits_{t = 1}^{J - 1} \Psi_{ij,t}^{2,g} }}{{\mathop \sum \nolimits_{j = 1}^{N} \mathop \sum \nolimits_{t = 1}^{J - 1} \Psi_{ij,t}^{2,g} }}$$

${\Pi }_{j,t}^{g} \left( J \right)$ can be interpreted as the variance share one variable has on others.^2^ The GFEVD verifies $\mathop \sum \limits_{j = 1}^{N} {\Pi }_{j,t}^{N} \left( J \right) = 1$ and $\mathop \sum \limits_{i,j = 1}^{N} {\Pi }_{j,t}^{N} \left( J \right) = N$.

Using the GFEVD, we can construct different connectedness indices. The first index is related to the *total connectedness,* which shows how a shock in one variable spills over to other variables and is defined by:

7 $$H_{t}^{g} \left( J \right) = \frac{{\mathop \sum \nolimits_{i,j = 1,i \ne j}^{N} \Pi_{ij,t}^{g} \left( J \right)}}{N} \times 100$$

Second, we calculated the directional connectedness that a variable $i$ receives from variables $j$, called *total directional connectedness from others*, defined as:

8 $$H_{i \leftarrow j,t}^{g} \left( J \right) = \frac{{\mathop \sum \nolimits_{i,j = 1,i \ne j}^{N} \Pi_{ij,t}^{g} \left( J \right)}}{{\mathop \sum \nolimits_{j = 1}^{N} \Pi_{ij,t}^{N} \left( J \right)}} \times 100$$

Similarly, we computed the directional connectedness that a variable $i$ transmits its shock to all other variables $j$, called *total directional connectedness to others*, defined by:

9 $$H_{i \to j,t}^{g} \left( J \right) = \frac{{\mathop \sum \nolimits_{i,j = 1,i \ne j}^{N} {\Pi }_{ji,t}^{g} \left( J \right)}}{{\mathop \sum \nolimits_{j = 1}^{N} {\Pi }_{ji,t}^{N} \left( J \right)}} \times 100$$

Finally, we defined the so-called *net total directional connectedness* as the difference between the two later indices:

10 $$H_{i,t}^{g} \left( J \right) = H_{i \to j,t}^{g} \left( J \right) - H_{i \leftarrow j,t}^{g} \left( J \right)$$

This index examines the ''power” of variable $i$, or its influence on the whole variables’ network. If $H_{i,t}^{g} \left( J \right) > 0$, the variable $i$ influences the network more than being influenced by it. If $H_{i,t}^{g} \left( J \right) < 0$, the variable $i$ is driven by the network.

#### EPU and dynamic connectedness between stock markets

Several recent studies argued that the relationship between financial markets, especially stock markets, is affected by economic policy uncertainty (Li et al. 2015; Li and Peng 2017; Fang et al. 2017, 2019; Badshah et al. 2019; Matkovskyy et al. 2020). Almost all these studies argue that EPU negatively impacts the correlations between these variables. Therefore, one can argue that economic policy uncertainty is a potential factor that drives the connectedness between the stock returns.

After computing the different time-varying spillover indices based on the TVP-VAR model, we examined whether economic policy uncertainty (EPU) drives this connectedness between stock market returns. To this end, we estimated the following equation:

11 $$H_{t} = \theta_{0} + \theta_{1} EPU_{t} + \varepsilon_{t}$$

where $H_{t}$ represents the total and net connectedness measures, as reported in Eqs. (7) and (10) between stock market returns. $EPU_{t}$ refers to changes in the US economic policy uncertainty index developed by Baker et al. (2016).

## Results and discussions

### Dynamic spillover results

Table 2 summarizes the estimates of the average dynamic connectedness measures for each stock market examined, generated by the TVP-VAR model. We observed that own-country stock market spillovers explained the highest share of forecast error variance, because the diagonal elements received higher values compared to the off-diagonal elements. Also, the total connectedness index (TCI) measured the average influence that all variables have on one variable's forecast error variance throughout time. The TCI in all markets was 65.43%, as shown in Table 2. This result indicates that international stock markets were not independent of each other; the average influence of a stock market was approximately 66%. This large value shows that the transmission of international stock market spillovers is an important source of domestic stock market fluctuation. Moreover, results showed that France contributed to the forecast error variance of all other examined markets by transmitting the highest index of 97.48%, followed by Germany 88.83%, Spain 84.55%, and Italy 82%. Further, these countries received the highest spillovers from others: France 77.9%, Germany 76.63%, and Spain and Italy around 75%. However, the contributions of China, Russia, the US, and the UK to others were the lowest, with 8.83%, 29.89%, 53.83%, and 77.32, respectively. Additionally, China, Russia, and the US received more spillovers than they transmitted, with 30.7%, 51.4%, and 61.76%, respectively. The bottom line in Table 2 presents the net spillovers for each country and shows that China, Russia, and the US were net receivers of spillover from all others. In contrast, all other examined countries were net transmitters for all others.

**Table 2:** Dynamic connectedness measures between stock returns

	China	France	Germany	Italy	Russia	Spain	UK	US	From
China	69.304	4.609	4.442	3.614	2.936	4.179	4.797	6.118	30.696
France	1.106	22.099	18.598	15.393	4.371	15.944	14.243	8.247	77.901
Germany	1.101	19.693	23.364	15.263	3.749	15.359	13.306	8.165	76.636
Italy	0.858	17.194	16.099	24.878	3.976	18.076	11.794	7.124	75.122
Russia	1.982	9.146	7.421	7.449	48.6	7.613	10.332	7.457	51.4
Spain	0.932	17.463	15.878	17.761	4.061	24.426	11.999	7.48	75.574
UK	1.344	16.886	14.854	12.518	5.752	13.083	26.322	9.242	73.678
USA	1.512	12.432	11.543	10.064	5.046	10.305	10.858	38.24	61.76
Contribution to others	8.835	97.423	88.835	82.063	29.892	84.559	77.327	53.833	522.767
Contribution including own	78.14	119.522	112.199	106.941	78.491	108.985	103.649	92.074	TCI
Net spillovers	-21.86	19.522	12.199	6.941	-21.50	8.985	3.649	-7.926	65.346

This table reports the variance decompositions for the estimated TVP-VAR model addressing different stock market returns. Variance decompositions are based on 10-step-ahead forecasts and a TVP-VAR lag length of order 1. The terms “Contribution to others” indicate the measure of the directional connectedness that a given variable i transmits its shock to all other variables j, following Eq. (9). The term “From” indicates the measure of the directional connectedness that a given variable i receives the shocks from all other variables j, following Eq. (8). “Net spillovers” means the difference between the two directional connectedness following Eq. (10). TCI indicates the total connectedness following Eq. (7)

**Fig. 2:**
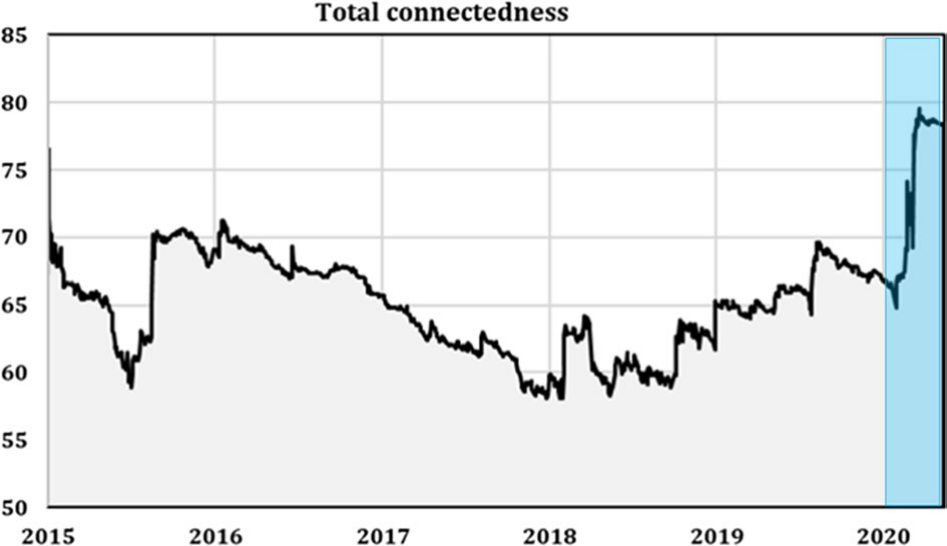
Total connectedness measure (the shaded blue area indicates the period of the COVID-19 pandemic)

To determine whether mean connectedness between stock markets varied through time and how it was affected by the COVID-19 pandemic, we estimated the different time-varying connectedness measures. Figure 2 presents the time path for the dynamic total connectedness index (TCI). We observed large variations in this index during the full sample period. Moreover, the total connectedness index was relatively high during the entire period. In mid-2015 and the first quarter of 2018, the TCI reached its lowest level, with approximately 57% and 55%, respectively. However, the total connectedness reached unprecedented heights during the COVID-19 pandemic outbreak in the first quarter of 2020, with a level close to 80%. These results are supported by Zhang et al. (2020a, b, c) and Cepoi (2020), who found that dependencies between stock markets increased remarkably during the health crisis outbreak. This result was not surprising in the sense that it is well known that stock markets are interlinked and interdependent (Morales and Andreosso 2012) and that crisis periods increase the global stock market's interdependence (Jondeau and Rockinger 2006; Mokni and Mansouri 2017). This result may be explained by the coronavirus pandemic and its rapid global spread reducing economic trends and inducing negative changes in investors’ sentiments that strongly affected their investment decisions, and consequently led to stock market price depreciations. Similarly, Wen et al. (2019) found that investor attention negatively predicted future stock prices’ crash risk. Such external and unexpected shock worldwide leaves investors more pessimistic about future returns and they consequently tend to take fewer risks. According to Bai (2014) and Baker et al. (2020a), investors may feel pessimistic about investment prospects in a given market, selling off that market's stocks under an infectious disease outbreak.

**Fig. 3:**
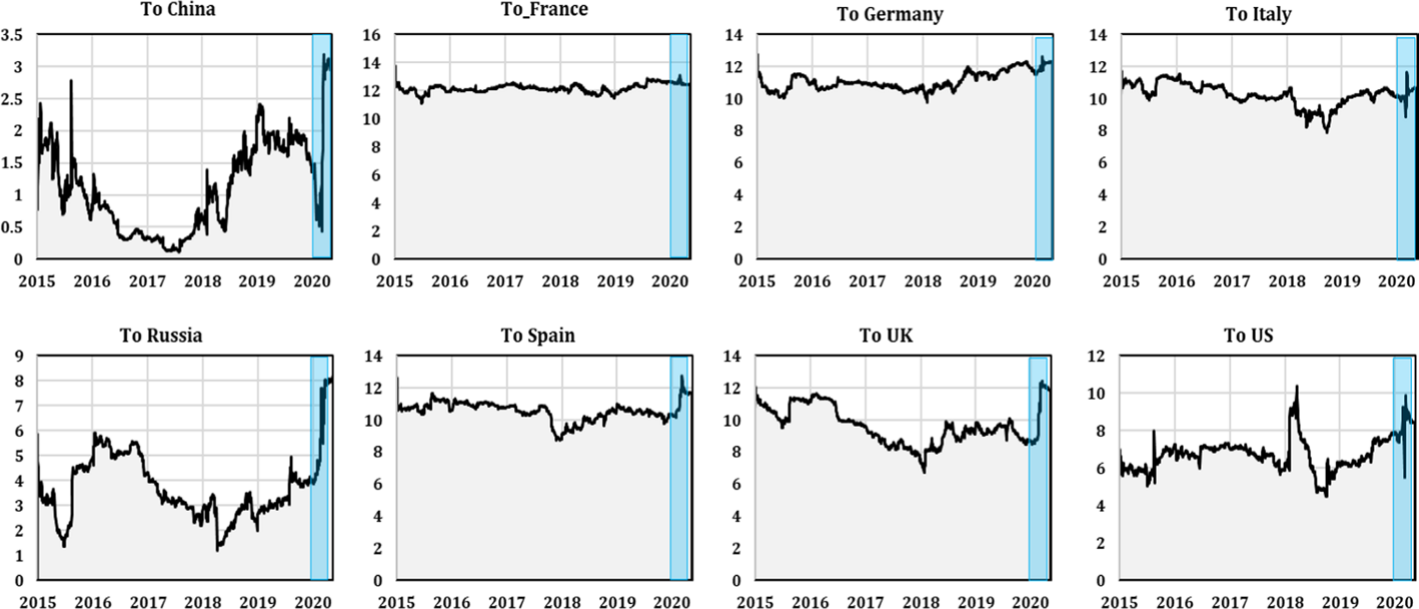
Dynamic spillover to the stock market in each country (the shaded blue area indicates the period of the COVID-19 pandemic)

**Fig. 4:**
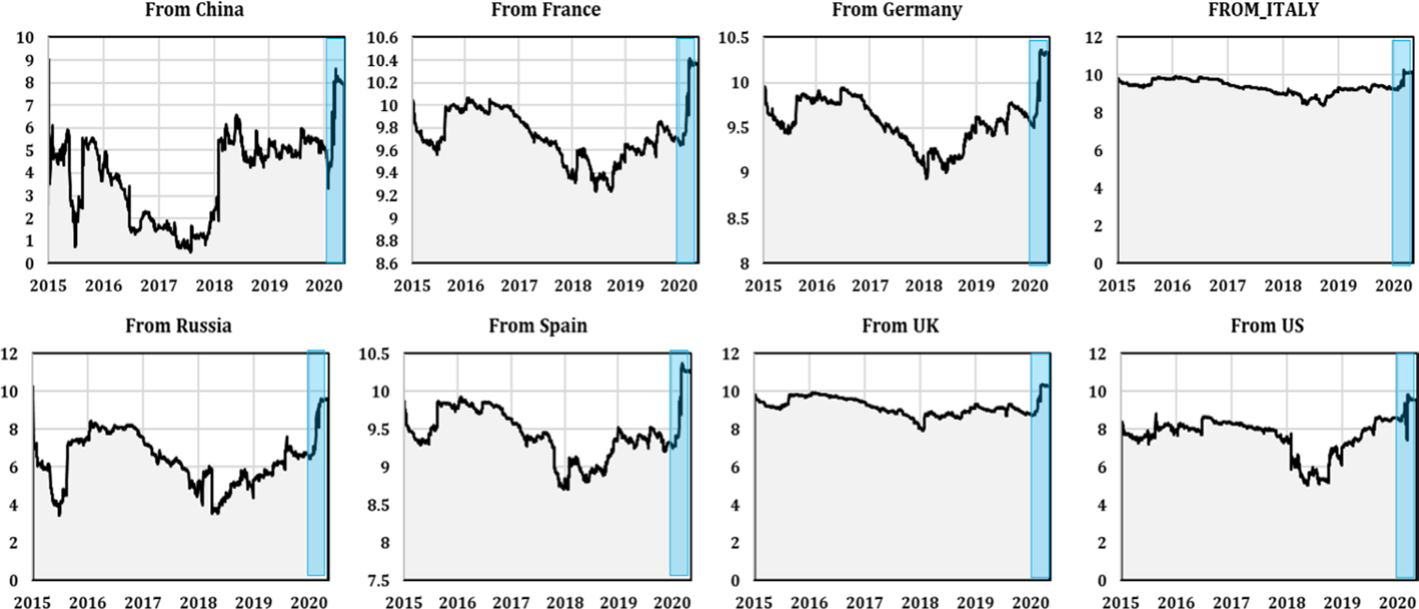
Dynamic spillover from the stock market in each country (the shaded blue area indicates the period of the COVID-19 pandemic)

This dynamic provides evidence that static TCI may mask specific periods (e.g., financial and economic events) that are likely to have different impacts on its interconnectedness. Therefore, it is crucial to consider the time-varying behavior of connectedness measures when analyzing the mechanism of transmission spillovers between stock markets to better understand the details of these spillovers, especially during critical periods. Therefore, we analyzed the total “to-directional” dynamic spillovers for each country and from all stock markets in each country, as shown in Figs. 3 and 4, respectively. These two figures show that the total dynamic spillovers from/to each series were bidirectional and ranged between 0.2 and 13%. Figure 3 depicts the amounts of spillover received by each country from all other countries. We observed that China, Russia, the UK, and the US showed significant time-varying transmission patterns. For China and Russia, the transmission of spillovers to these countries’ stock markets from all others increased significantly during the first quarter of 2020, which coincided with the outbreak of the COVID-19 pandemic, reaching a peak of 3.2% and 8% in China and Russia, respectively. For the UK and the US, a weak dynamic transmission pattern was observed compared to China and Russia, which had high spillover levels ranging between 6 and 12% from all others to the UK and the US, but these spillovers were less dynamic.

We found that Euro area zone countries' stock markets were less affected by the transmission of spillovers from all others. For France, the transmission patterns were almost static during the spanning period, with no change in the transmission of spillovers to France’s stock market during the COVID-19 pandemic outbreak. For Germany, Italy, and Spain, the total dynamic spillovers to stock markets were significant and, to an extent, time-varying compared to France. However, similar to the French stock market, the total dynamic spillovers to Germany, Italy, and Spain stock markets from all others did not vary during the pandemic period. These results may have occurred because, following China, the pandemic broke out in Europe and dampened economic activity. Consequently, European countries experienced downturns after the pandemic’s announcement in the international media. Thus, stock markets were suffering a large decline and no longer reacted to bad news related to the recent health crisis or to different countries' policies in response to the rapid spread of COVID-19.

Figure 4 depicts the spillover levels transmitted by each country to all other stock markets. China, Russia, France, Germany, and Spain transmitted the most to all other stock markets. In these countries, the dynamic transmission patterns were significantly time-varying, with increased transmission of spillovers to all others, especially during the COVID-19 pandemic outbreak. For China, the transmission of spillovers jumped from 4% before the pandemic onset to nearly 9% during the pandemic outbreak. The dynamic transmission of spillovers from Russia to all other stock markets also increased during the pandemic period, jumping from 7% before the COVID-19 pandemic period to nearly 10% during the pandemic period.

The dynamic spillovers from France, Germany, and Spain behaved heterogeneously over time and followed a transmission pattern similar to all other stock markets. Moreover, we registered increased dynamic spillovers from these countries during the COVID-19 pandemic period, which jumped from an average of 9.5% before the outbreak to nearly 10.5% after the outbreak. The total directional spillovers from Italy to all stock markets were almost static and did not vary during the spanned period. The total directional spillovers from the UK and the US to all others were less dynamic and rather static over time, with weak variations during the sample period. Nevertheless, the dynamic spillovers from these countries increased slightly during the pandemic period and increased from 9% (7%) for the UK (US) before the pandemic outbreak to close to 10% for both after the outbreak.

Comparing the dynamic spillover results, we found that the European stock markets transmitted more spillovers to all other stock markets (except Italy) than they received. This role became more pronounced during the COVID-19 outbreak. A plausible explanation is that the worldwide announcement of the pandemic may have changed the way market participants perceived risk and they may have begun to expect higher levels of bad shock transmission. Therefore, markets under pressure tend to transmit risks more than in normal periods, leaving investors less confident about predicting risks, which in turn increases spillovers from bad news (shocks).

**Fig. 5:**
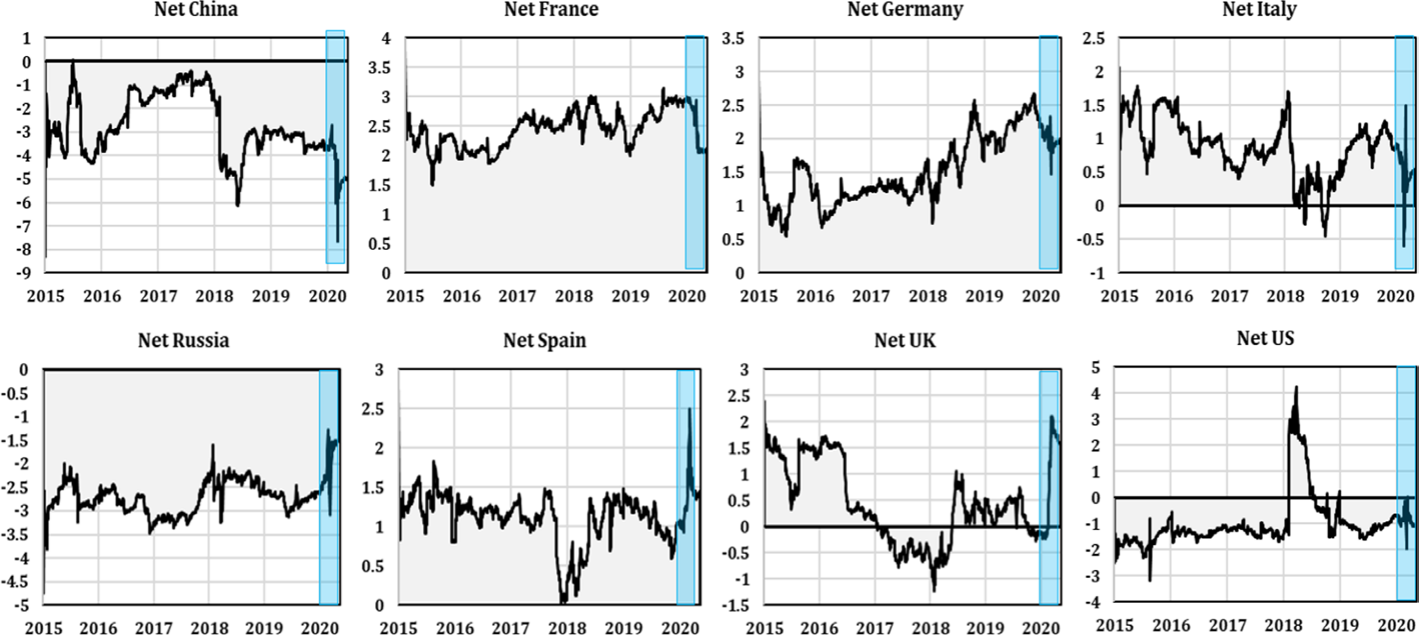
Net dynamic spillover of the stock market in each country (the shaded blue area indicates the period of the COVID-19 pandemic)

A similar picture emerged in the net dynamic total directional connectedness, depicted in Fig. 5. China and Russia were mostly net receivers of spillovers during the sample period. The US acted as a net transmitter of spillovers during the first half 2018, but a net receiver of spillovers during the remaining periods. Finally, the UK acted as a net receiver of spillovers from other countries from 2017 to the first quarter of 2018, then became a transmitter during the remaining periods. These results may have stemmed from the US market being one of the main sources of a spillover effects on other markets and regions (Bekaert et al. 2011; Syriopoulos et al. 2015). Whereas the rapid spread of the coronavirus to the US and the UK following China, South Korea, Iran, and Italy, remarkably increased the geopolitical risk index, and uncertainty about COVID-19′s short- and long-term consequences became the main concern of US policymakers (Sharif et al. 2020), which turned the US market into a net receiver of spillovers from others. Another explanation is the conduction mode of the COVID-19 shock and its causes. Unlike the GFC that began in the US and was caused by different structural problems in the US economy, COVID-19 had a single unique cause, the spread of the coronavirus following its discovery in China, and there were no other typical early warning signals of an impending financial crisis (Yarovaya et al. 2020). Therefore, the spillover dynamics from and to other countries may differ from those of previous financial crises.

The Euro area zone countries were net transmitters of spillovers to all other countries. These findings are in line with Zhang et al.’s (2020a, b, c), who found that the European stock markets were strongly connected during the pandemic outbreak and that France and Germany were in the core of the minimum spanning tree. Moreover, the authors showed that the US and mainland China stock markets were isolated from the system before the pandemic announcement. After the announcement, Asian stock markets, which were scattered before the announcement, formed a cluster, and became more integrated. Both before the pandemic announcement and after, the US stock market failed to take a leading role.

Recently, the financial press highlighted that, compared to the GFC, the COVID-19 pandemic created an enormous uncertainty shock—larger than the shock associated with the financial crisis of 2008–2009 and more similar in magnitude to the rise in uncertainty during the Great Depression of 1929–1933. In fact, the greatest impact of the 2008 global financial crisis—which had strong economic destructive power and caused a huge chain reaction, destroying the financial industry—was that it allowed systemic risk to be regarded as a crucial factor in financial safety. Even today, the global economy has not fully recovered from the aftermath of these events that derived from the destructive effects of systemic financial risk (Kou et al. 2019). Moreover, comparing our results to findings on the GFC’s effects on stock market interconnectedness, it is clear that the two crises created similar effects. Our results are consistent with Gong et al. (2019) and Zhang et al. (2020a, b, c), who found that Lehman Brothers’ collapse drove the connectedness jump to its highest level. The liquidity crisis of August 2007 plunged the European stock market into a panic, which resulted in large investment write-offs in US mortgage-backed assets. This strengthened the connection between the US and European stock markets. In the same context, our findings are consistent with BenSaida (2019), who found that the US did not play a leading role in volatility transmission from the traditional spillover measures. Still, its contribution of bad shocks substantially increased during the GFC, which was born in the US, repositioning it as a leading bad risk transmitter.

Similarly, French and Italian contributions to the transmission of bad shocks increased during the European sovereign debt crisis that started in Greece at the end of 2009 and was soon followed by Italy in 2010. These results support our findings that, under pressure, the market tends to transmit high volatility spillovers. Similarly, our findings are in line with Liu et al. (2017), who investigated the spillover networks between the G20 countries’ stock markets. Their findings confirmed that obvious volatility spillovers exist and are changing over time, and that G20 markets are closely connected. They found that the US ranked second, after Korea, for sending volatility spillover to other countries during the subprime crisis period.

Conversely, our findings contradict the research mentioned above regarding the US's leading role in the economic system. We found that, during the recent COVID-19 pandemic, the US acted as a recipient of spillovers from other countries. As explained earlier, some countries changed roles from net transmitters to net receivers of spillovers, which may have resulted from event-driven and time-varying spillover effects and other factors. The main factor may be attributed to the crisis' conduction mode, economy characteristics, and geographic proximity that may influence a country's role in absorbing risk or transmitting risk to other countries or regions. Zhang et al. (2020a, b, c) found that countries relying heavily on foreign direct investments were more likely to absorb risks during a subprime crisis, while countries and regions with high foreign portfolio holdings were more inclined to risk spillovers.

### Asymmetric connectedness results

To provide more detailed information on dynamic connectedness, we extended our analysis to a nonlinear framework to control whether the connection differed across increases and decreases in stock indices. We separated the positive and negative variations from the different returns series and re-estimated the dynamic spillovers between the examined stock markets. We then separated the dynamic connectedness of the positive returns from the negative returns. Therefore, we defined the positive and negative returns as: $R_{t}^{ + } = {\text{max}}\left( {R_{t} ,{ }0} \right)$ and $R_{t}^{ - } = {\text{min}}\left( {R_{t} ,{ }0} \right)$, verifying $R_{t} = R_{t}^{ + } + R_{t}^{ - }$, where $R_{t}$ denotes the stock returns of a given country at time t.

The average of the different connectedness indices based on the TVP-VAR(1) for the positive and negative stock return variations are reported in Table 3. The total connectedness index (TCI) for the negative returns was higher than for the positive returns, indicating that negative spillover effects had more substantial magnitudes than positive ones. In other words, the bad news in stock markets, expressed by negative returns, was transmitted more intensively than the good news expressed by positive returns, and had an asymmetric spillover transmission globally. Moreover, different stock markets retained the same character regarding the net receiver of spillovers transmitter effects for positive and negative returns. China, Russia, the UK, and the US stock markets were still net receivers of both good and bad news, while other markets were net transmitters.

**Table 3:** Dynamic asymmetric connectedness table

	China	France	Germany	Italy	Russia	Spain	UK	US	From
*Panel A: Positive returns*									
China	73.790	3.765	4.183	2.931	2.865	2.777	3.991	5.697	26.210
France	0.984	24.902	19.167	15.079	4.416	15.267	13.686	6.498	75.098
Germany	1.072	20.275	26.374	14.917	3.770	14.712	12.118	6.761	73.626
Italy	0.793	16.955	15.848	28.401	3.710	17.779	10.847	5.668	71.599
Russia	2.151	8.985	7.417	6.978	51.670	7.247	8.735	6.817	48.330
Spain	0.851	16.932	15.415	17.474	3.787	27.898	11.592	6.052	72.102
UK	1.462	16.544	13.757	11.745	4.926	12.714	30.848	8.004	69.152
US	2.012	10.553	10.107	8.539	4.778	8.840	10.076	45.096	54.904
Contribution to others	9.325	94.009	85.894	77.662	28.253	79.336	71.046	45.498	491.022
Contribution including own	83.115	118.911	112.268	106.062	79.923	107.234	101.894	90.594	TCI
Net spillovers	−16.885	18.911	12.268	6.062	−20.077	7.234	1.894	−9.406	61.378
*Panel B: Negative returns*									
China	58.245	6.318	5.665	5.556	4.206	5.376	5.790	8.845	41.755
France	1.506	22.247	18.597	15.025	3.924	15.889	13.499	9.313	77.753
Germany	1.561	19.780	23.388	15.017	3.425	14.997	12.753	9.079	76.612
Italy	1.308	16.978	16.032	24.932	3.664	18.013	10.738	8.335	75.068
Russia	2.576	8.487	6.876	7.115	50.688	7.735	8.945	7.578	49.312
Spain	1.130	17.618	15.566	17.547	3.869	24.649	11.087	8.535	75.351
UK	1.611	16.898	15.053	12.005	5.138	12.705	26.266	10.324	73.734
US	2.048	12.815	12.021	10.600	4.409	10.657	9.992	37.459	62.541
Contribution to others	11.740	98.894	89.809	82.864	28.634	85.372	72.805	62.009	532.126
Contribution including own	69.985	121.141	113.197	107.796	79.322	110.021	99.071	99.468	TCI
Net spillovers	−30.015	21.141	13.197	7.796	−20.678	10.021	−0.929	−0.532	66.516

This table reports the variance decompositions for the estimated TVP-VAR model addressing different stock market returns. Variance decompositions are based on 10-step-ahead forecasts and a TVP-VAR lag length of order 1. The terms “Contribution to others” indicate the measure of the directional connectedness that a given variable i transmits its shock to all other variables j, following Eq. (9). The term “From” indicates the directional connectedness measure that a given variable i receives the shocks from all other variables j, following Eq. (8). “Net spillovers” means the difference between the two directional connectedness following Eq. (10). TCI indicates the total connectedness following Eq. (7)

**Fig. 6:**
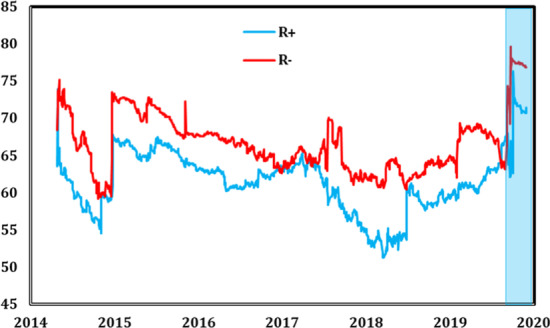
Total dynamic spillover of positive (R +) and negative (R-) stock returns (the shaded blue area indicates the period of the COVID-19 pandemic)

**Fig. 7:**
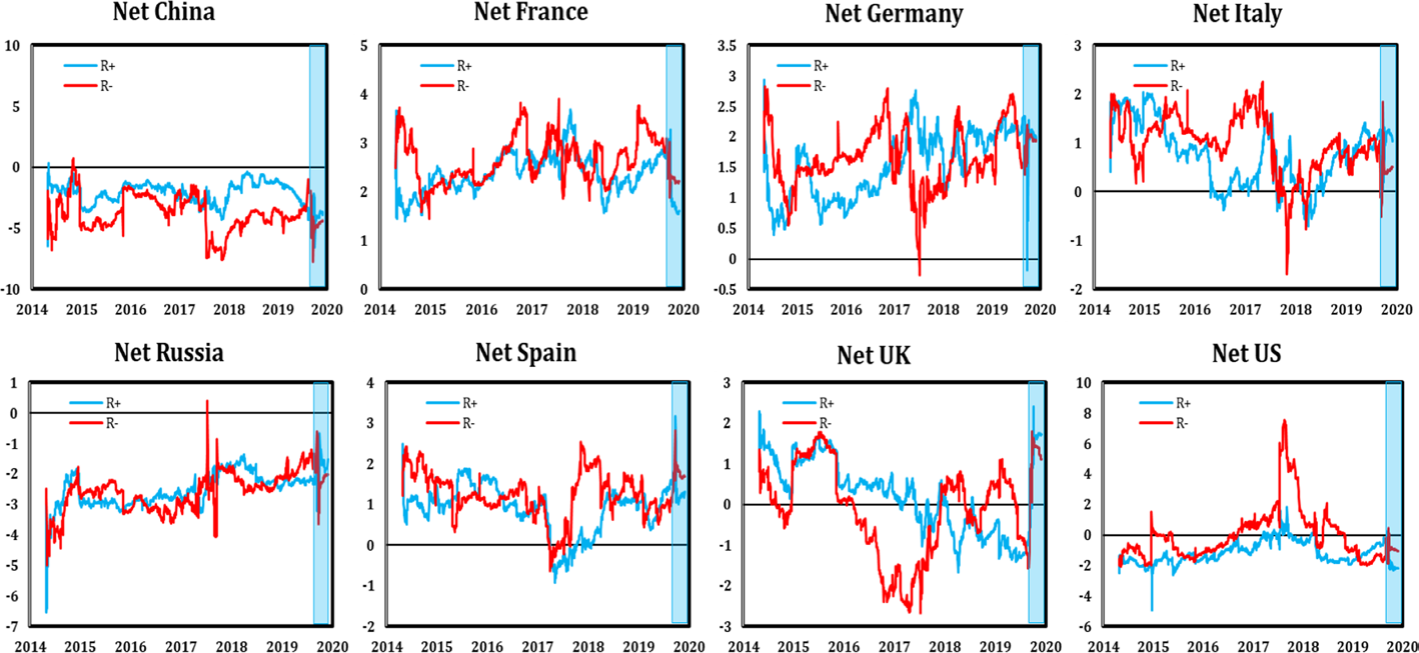
Net dynamic spillover of positive (R +) and negative (R-) stock returns (the shaded blue area indicates the period of the COVID-19 pandemic)

Providing a more detailed picture of the evolution of the different dynamic connectedness, Figs. 6 and 7 depict the total and net connectedness indices, respectively. The evolution of the total connectedness index indicates that both positive and negative shocks were transmitted at different magnitudes that changed over time, reaching max values over the first quarter of 2020 in conjunction with the COVID-19 pandemic period. Moreover, comparisons of the positive and negative returns’ connectedness showed ample evidence of asymmetric connectedness, justified by a different pattern of negative and positive shocks transmission. The level of total connectedness for negative variations was more intensive than for positive variations over most of the study period.

Figure 7 plots the asymmetric net connectedness between stock markets for the case of positive and negative returns. The results showed that the Chinese and Russian stock markets were net receivers of spillover effects during most periods, while in the UK and US stock markets, the net spillovers index switched between positive and negative, indicating that these advanced markets had a changeable character regarding shock transmission. By contrast, the other European countries generally acted as net transmitters of both positive and negative shocks with French market dominance being remarkable.

By focusing on the COVID-19 pandemic period, similar results were observed in the symmetric spillover analysis shown in Fig. 2. We observed an exceptional increase in the total connectedness for both positive and negative returns, indicating that the different stock markets were more interconnected during the COVID-19 outbreak.

### Dynamic spillover and EPU

The rapid spread of COVID-19 to countries worldwide caused unprecedented effects on global financial stock market risks and substantially increased geopolitical risks and economic policy uncertainty (EPU). We investigated the effect of EPU on the connectedness between the eight examined stock markets. For comparison, we investigated the effect of the EPU during the entire period before and during the COVID-19 pandemic. The sample period was divided into two sub-periods, with the first spanning from 01/01/2015 to 12/23/2019 (pre-COVID-19 outbreak), and the second spanning from 12/24/2019 to 05/18/2020, covering the COVID-19 outbreak period. We chose the second period start date because it coincided with the beginning of frequent news about the emerging pandemic. During this period, the Wuhan Municipal Health Commission in China reported a cluster of cases of a novel unidentified coronavirus. Given that the financial markets are sensitive to this type of news (Baker et al. 2020a, b; Ramelli and Wagner 2020; Phan and Narayan 2020; Azimli 2020), by starting with this date we covered the maximum period of the COVID-19 pandemic.

Table 4 reports estimates of the EPU’s effect on total and net connectedness for the examined stock markets, as described in Eq. (11). Panel (A) reports the effect of the EPU during the entire sample period. Results showed that the EPU’s effect on total connectedness was significantly positive. Also, EPU’s effect on net connectedness for each country was significant except for France and the US. Moreover, Chinese and Italian net connectedness were negatively affected by the EPU, while a positive connectedness was reported for all other markets.

**Table 4:** Effect of the EPU on the connectedness between the stock market before and after COVID-19 pandemic

	Total connect	Net connectedness							
		China	France	Germany	Italy	Russia	Spain	UK	US
*Panel A: Full period*									
$\theta _{0}$	63.0278***	−2.3180***	2.4566***	1.3867***	0.9731***	−2.8318***	1.0697***	0.3039***	−1.0401***
	(0.1716)	(0.0626)	(0.0151)	(0.0233)	(0.0200)	(0.0173)	(0.0158)	(0.0363)	(0.0479)
$\theta _{1}$	0.0239***	−0.0042***	−0.0002	0.0014***	−0.0011***	0.0015***	0.0006***	0.0016***	0.0005
	(0.0013)	(0.0005)	(0.0001)	(0.0002)	(0.0002)	(0.0001)	(0.0001)	(0.0003)	(0.0004)
*Panel B: Before COVID-19*									
$\theta _{0}$	64.9779***	−2.6990***	2.3216***	1.2773***	0.9872***	−2.6534***	1.1577***	0.6811***	−1.0726***
	(0.2402)	(0.0898)	(0.0212)	(0.0339)	(0.0293)	(0.0231)	(0.0224)	(0.0512)	(0.0717)
$\theta _{1}$	−0.0027	0.0012	0.0014***	0.0026***	−0.0012***	−0.0010***	−0.0007***	−0.0032***	0.0008
	(0.0026	(0.0010)	(0.0002)	(0.0004)	(0.0003)	(0.0002)	(0.0002)	(0.0006)	(0.0008)
*Panel C: COVID−19 period*									
$\theta _{0}$	65.9934***	−3.7050***	2.9934***	2.1246***	0.6737***	−2.4235***	1.2975***	−0.1945**	−0.7658***
	(0.4656	(0.1382)	(0.0320)	(0.0242)	(0.0761)	(0.0791)	(0.0684)	(0.0920)	(0.0685)
$\theta _{1}$	0.0241***	−0.0029***	−0.0017***	−0.0004***	−0.0005**	0.0014***	0.0004**	0.0038***	−0.0002
	(0.0013)	(0.0004)	(0.0001)	(0.0001)	(0.0002)	(0.0002)	(0.0002)	(0.0002)	(0.0002)

This table provides the parameter estimates of the model in Eq. (11). Numbers between parenthesis denote the estimated standard error. (***), (**), and (*) indicate the parameters significance at 1%, 5%,, and 10%, respectively

Panels (B) and (C) report the EPU effects on total and net connectedness before and during the COVID-19 pandemic onset. Results showed that the EPU’s effect on total connectedness was insignificant before the pandemic outbreak but became significantly positive following the pandemic announcement. This result indicates that global stock markets tend to collectively move in the same direction during periods of pressure and high economic uncertainty. Thus, what is good for one market is also good for all other markets and vice versa.

The EPU had a significant effect on all stock markets except China and the US before the COVID-19 pandemic onset. EPU positively affected net connectedness for the France and Germany stock markets while negatively affecting the Italy, Spain, Russia, and UK stock markets. Within the COVID-19 pandemic outbreak, results were slightly different. Except for the US, all examined stock markets affected by EPU showed significant net connectedness resulting from the pandemic outbreak. In addition, the direction of the EPU’s effect changed before and during the outbreak. The EPU’s effect on the net connectedness of the France and Germany stock markets turned significantly negative, but the effect was significantly positive for Russia, Spain, and the UK. However, the Italian stock market’s net connectedness was significantly negative before and during the COVID-19 pandemic onset. These results underscore the strong link between economic policy uncertainty and stock market net connectedness in countries worldwide.

The direction change in the EPU’s effect on net connectedness before and during the pandemic onset indicate that information spillovers from a given market may manifest as either good or bad news for other markets, given the prevailing economic situation. For example, under the normal economic situation (before the COVID-19 outbreak), net connectedness from China’s stock markets did not respond to economic uncertainty. In contrast, with the onset of the pandemic (a high economic uncertainty state), the bad news spillovers from these stock markets to other markets were significantly affected by the prevailing high economic uncertainty. The direction change of the EPU effects indicate that dynamic transmission of spillovers from a given market depend on whether the economic state is normal (low levels of uncertainty) or under pressure (high uncertainty level). Thus, bad news from China during the COVID-19 pandemic outbreak and the policies adopted by Chinese authorities in reaction to the new pandemic could be either bad or good news to other countries around the world.

Conversely, Europe was the second center of coronavirus spread, especially Italy, followed by Spain, France, and Germany. In this short period, European countries had little experience with reacting to pandemic spread and did not yet have plans and policies they could implement to protect their economies and stock markets. Therefore, bad news coming from China was bad news for European stock markets. While other countries that were affected later, such as the US, the UK, and Russia, were better informed about the pandemic spread risk. Consequently, these countries profited from the time lag and other countries' experiences in mitigating the risk.

### Robustness analysis

To verify our results, we conducted a robustness analysis by changing the VAR lag orders,$k,$ from one to five days.^3^ Table 5 presents the estimates of the total and net connectedness means measured based on the different lag orders $k$.^4^

**Table 5:** Total and net dynamic connectedness averages based on different TVP-VAR lag orders

	Net spillovers								TCI
	China	France	Germany	Italy	Russia	Spain	UK	US	
*Returns*									
$k = 1$	−21.86	19.522	12.199	6.941	−21.509	8.985	3.649	−7.926	65.346
$k = 2$	−22.16	19.457	11.983	7.568	−21.682	9.451	3.992	−8.609	65.747
$k = 3$	−24.241	19.847	12.754	7.951	−21.72	8.693	1.682	−4.966	66.033
$k = 4$	−24.888	20.192	12.572	8.018	−21.259	8.779	2.696	−6.111	66.646
$k = 5$	−27.521	20.388	13.076	8.267	−20.806	9.377	3.879	−6.661	67.491
*Positive returns*									
$k = 1$	−16.885	18.911	12.268	6.062	−20.077	7.234	1.894	−9.406	61.378
$k = 2$	−18.905	19.165	11.719	6.899	−20.444	7.741	3.573	−9.748	62.452
$k = 3$	−13.386	18.667	10.69	4.896	−19.964	7.244	0.301	−8.447	59.999
$k = 4$	−16.58	18.404	10.899	5.715	−19.541	7.83	0.561	−7.288	60.907
$k = 5$	−17.39	18.053	10.441	6.335	−20.001	8.289	0.754	−6.481	61.869
*Negative returns*									
$k = 1$	−30.015	21.141	13.197	7.796	−20.678	10.021	−0.929	−0.532	66.516
$k = 2$	−27.667	20.847	13.23	8.035	−21.479	9.496	0.121	−2.582	66.565
$k = 3$	−28.208	19.29	10.69	9.633	−16.894	9.068	−2.017	−1.561	63.988
$k = 4$	−27.422	18.559	11.209	10.035	−17.457	9.475	−1.783	−2.616	64.428
$k = 5$	−29.064	18.888	11.153	10.673	−18.446	9.631	−0.651	−2.185	65.341

This table contains the averages of the dynamic total and net spillover indices based on the TVP-VAR model with lag order between 1 and 5

With the TVP-VAR(1) results as a benchmark, as shown in Tables 2 and 3, the means of the different connectedness indices were similar to those resulting from the TVP-VAR(1) model for all examined markets. The total connectedness index varied between 65 and 67% for all returns, between 59 and 61% for the positive returns, and between 63 and 66% for the negative returns. Similar results were obtained for the net connectedness index.

## Conclusion and policy implications

The effects of a public health emergency can be transmitted to the economy because stock markets serve as a barometer of investors’ expectations and faith in economic prospects (Bai 2014; Baker et al. 2012). The COVID-19 pandemic spread intensified uncertainties worldwide, increasing stock investors’ fear, and creating pessimistic sentiments regarding future returns. This study analyzed the dynamic connectedness between the major stock markets affected by the coronavirus pandemic. Moreover, we analyzed the effect of economic policy uncertainty on the dynamic directional connectedness between stock markets before and during the COVID-19 pandemic onset by applying the TVP-VAR model recently proposed by Antonakakis and Gabauer (2017). This methodology substantially improves on Diebold and Yilmaz’s (2014) connectedness approach. It also overcomes the limitations of the often arbitrarily chosen rolling-window-size, which could lead to very erratic or flattened parameters as well as loss of valuable observations.

Analyzing the eight stock markets’ daily data, the results revealed an unprecedented sensitivity of stock market connectedness to the rapid spread of the COVID-19 pandemic. Results also showed that total connectedness between stock markets and net connectedness within each stock market varied over time, depending on the economic uncertainty state.

Our findings reveal different regularities. First, the total dynamic connectedness between the examined stock markets substantially increased and reached unprecedented levels during the COVID-19 pandemic outbreak. This result confirms previous findings suggesting that stock market links become more pronounced during crisis periods. According to Gormsen and Koijen (2020), such dramatic movement can occur because, under long-term expectations, it is almost certain that sentimental factors play an important role. Broadstock and Zhang (2019) showed that market sentiment in response to the outbreak is quickly amplified through social media, which then stimulates trade activities and causes extreme price movements.

Second, results showed that China and Russia acted as net receivers of dynamic spillovers during the entire sample period. The US stock market acted as a net receiver for most of the period. The European stock markets were net transmitters of spillovers for all other markets, and the US stock market acted mostly as a transmitter. Furthermore, we found that, during the coronavirus pandemic outbreak, European stock markets, except Italy and the UK, transmitted spillovers more than they received them. This suggests that, in crisis periods, stock markets are more likely to transmit risks (bad volatility). Negative spillovers suggest that uninformed traders dominate the system, and bad spillover tends to transmit at a larger magnitude (BenSaida 2019).

Third, our study was extended to a nonlinear framework by separating positive returns from negative return to control for possible differences in good and bad news dynamic spillovers. The results showed an asymmetric dynamic spillover between stock markets, highlighted by more pronounced spillover effects for negative than for positive returns. Moreover, the results suggest that the dynamic spillovers were intensified during the COVID-19 pandemic, indicating that the pandemic caused a high level of dynamic spillovers for both positive and negative returns.

Fourth, we analyzed the effect of economic policy uncertainty (EPU) on the total directional connectedness and the net connectedness for each stock market. Results showed a significant positive effect of EPU on the total dynamic spillover during the entire sample period and during the coronavirus outbreak. Results showed different regularities regarding net connectedness for each stock market. First, over the full period, EPU significantly impacted net connectedness for all examined stock markets except France and the US. To further analyze the EPU’s effect on net connectedness during the COVID-19 outbreak, we divided the sample period into two sub-periods covering before and during the pandemic outbreak. Comparing the results for each sub-period, we found that: (i) before the pandemic outbreak, each stock market's net connectedness was significantly affected by EPU for all countries except China and the US; during the pandemic outbreak, all stock markets' net connectedness was significantly affected by EPU. (ii) The EPU’s effect on net connectedness, before and during the COVID-19 outbreak, switched between negative and positive. Thus, what was good for a given market may be either bad or good for other markets. (iii) The changing direction of the EPU’s effect on net connectedness, before and during the onset of COVID-19 suggests that stock markets’ response to news coming from a given market depends on the economic uncertainty state. Thus, during the COVID-19 outbreak, bad news from China was bad news for some countries that were rapidly affected by the new virus (Italy, Spain, and France). However, some other countries formerly affected by the new coronavirus were less sensitive to bad news coming from other countries, because authorities had already implemented new policies to alleviate the impact on their economies and stock markets of bad news related to coronavirus spread.

These findings have policy implications. The rapid spread of the new COVID-19 pandemic caused a high level of dynamic connectedness between international stock markets, an unprecedented shutdown of stock market returns, and increasing economic uncertainty worldwide. Therefore authorities, central banks, and investment banks must implement efficient economic strategies and policies to manage the COVID-19 crisis without triggering uncertainty. Similarly, government interventions should focus on alleviating the financial stock markets' panic mode and increasing investors' confidence in future revenues and market recovery. In addition, given the continuous spread of COVID-19 worldwide, market participants and investors should learn to manage stock market risk and panic.

Further, our findings provide insight for risk regulators, who should improve risk early warning systems by establishing a daily monitoring mechanism for the international transmission of financial risks. They should implement management systems that manifest the use of advantages, prudent prevention, and risk control. Also, countries should establish a comprehensive evaluation index system with rapid data updating and high transparency, covering various markets and industries (Zhang et al. 2020a, b, c).

Finally, it is important to strengthen international cooperation among financial regulators worldwide. In the context of economic globalization, stock markets are a complex economic ecosystem, so it is necessary to reinforce international sharing of management information related to financial risk contagion. Given that preventing international financial risk contagion is a long-term process, it is very important to implement a long-term risk governance system combined with global forces to manage early risk warning and recovery (Zhang et al. 2020a, b, c).

As with all studies, our research has some limitations. Because of the short event period and the virus's evolving nature, we could only study the immediate and short-term effects of the COVID-19 outbreak on the dynamic connectedness between the major affected countries’ stock markets. Future research should consider the pandemic’s long-term effects on stock markets’ connectedness, investor confidence inside and between foreign stock markets, and investor sentiment and uncertainty.

## Data Availability

Data will be made available upon request.

## Footnotes

1 The economic policy uncertainty index used in our study is a daily news-based index. It is related to the search results from newspaper archives from Access World New's NewsBank service. For more details, please see Baker et al. (2016) or the website: https://www.policyuncertainty.com

2 These variance shares are normalized in the sense that each row sums to 1. This means that all variables together explain 100% of *variable* i *'s* forecast error variance (Antonakakis and Gabauer 2017).

3 In addition to the lag order, we also established a robustness analysis based on several values of the variance forecasting horizon. We found very similar spillover measures and no significant differences were detected. Given space limitations, the results are available upon request.

4 To gauge the robustness of the results, we also plotted the different dynamic spillover indices to compare the evolution of each one with those of the original model in Figs. 2, 3, 4, 5, 6, 7. These figures, which are available upon request because of space limitations, show clear similarities with those found based on the original model.
